# Critical Care Database Comprising Patients With Infection

**DOI:** 10.3389/fpubh.2022.852410

**Published:** 2022-03-17

**Authors:** Ping Xu, Lin Chen, Yuanfang Zhu, Shuai Yu, Rangui Chen, Wenbin Huang, Fuli Wu, Zhongheng Zhang

**Affiliations:** ^1^Emergency Department, Zigong Fourth People's Hospital, Zigong, China; ^2^Artificial Intelligence Key Laboratory of Sichuan Province, Zigong, China; ^3^Institute of Medical Big Data, Zigong Academy of Artificial Intelligence and Big Data for Medical Science, Sichuan, China; ^4^Department of Critical Care Medicine, Affiliated Jinhua Hospital, Zhejiang University School of Medicine, Jinhua, China; ^5^Key Laboratory of Emergency and Trauma of Ministry of Education, Hainan Provincial Key Laboratory for Tropical Cardiovascular Diseases Research, The First Affiliated Hospital of Hainan Medical University, Research Unit of Island Emergency Medicine of Chinese Academy of Medical Sciences, Hainan Medical University, Haikou, China; ^6^Department of Health Management Center, Zigong Fourth People's Hospital, Zigong, China; ^7^Department of Gynecology, Fushun County Maternal and Child Health Hospital, Fushun, China; ^8^Department of Obstetrics, Fushun County Maternal and Child Health Hospital, Fushun, China; ^9^Department of Emergency Medicine, Sir Run Run Shaw Hospital, Zhejiang University School of Medicine, Hangzhou, China; ^10^Key Laboratory of Precision Medicine in Diagnosis and Monitoring Research of Zhejiang Province, Hangzhou, China

**Keywords:** critical care, database, open access, infections, big data and analytics

## Abstract

Patients treated in the intensive care unit (ICU) are closely monitored and receive intensive treatment. Such aggressive monitoring and treatment will generate high-granularity data from both electronic healthcare records and nursing charts. These data not only provide infrastructure for daily clinical practice but also can help to inform clinical studies. It is technically challenging to integrate and cleanse medical data from a variety of sources. Although there are several open-access critical care databases from western countries, there is a lack of this kind of database for Chinese adult patients. We established a critical care database involving patients with infection. A large proportion of these patients have sepsis and/or septic shock. High-granularity data comprising laboratory findings, baseline characteristics, medications, international statistical classification of diseases (ICD) code, nursing charts, and follow-up results were integrated to generate a comprehensive database. The database can be utilized for a variety of clinical studies. The dataset is fully accessible at PhysioNet(https://physionet.org/content/icu-infection-zigong-fourth/1.0/).

## Background and Summary

Infection is common in the intensive care unit (1, 2). There are two categories of infections for patients in the intensive care unit (ICU) due to the place where the infection was acquired. One type of infection is the infection present on ICU admission, and most of such patients are transferred to ICU due to the development of sepsis and/or septic shock (3, 4). The other type of infection is the infection acquired after ICU admission, which is also termed the nosocomial infection (1). Critically ill patients are at increased risk of infection because of compromised immunity, use of intravascular catheters, and endotracheal intubation (5, 6). Irrespective of the places where the infection is acquired, the infection can cause systematic inflammatory response (SIRS), sepsis, and septic shock. These complications are associated with a significantly increased risk of mortality (7, 8). Although sepsis has been widely investigated in the literature (4, 9, 10), the raw data are typically not publicly available due to confidential or legal issues. The restricted data usage created a barrier to reproducing and verifying the results.

Although several open-access critical care databases from western countries have been created to promote data sharing and reuse for the scientific community (11–15), there is a lack of such database comprising Chinese adult patients. Since the Chinese population is the largest in the world, exploring infection/sepsis in the Chinese population is the key to achieving the goal proposed by the surviving sepsis campaign (16). Furthermore, a dataset, especially those generated from electronic healthcare records is large in volume. Secondary analysis of such dataset can generate novel insights into the diseases of interest (13, 17–19). Thus, creating a critical care database relating to patients with infection can help to promote collaborative research across the globe to reveal more insights into the infections in critically ill patients.

The rationales to include all critical patients with infection are 2 folds. First, such a database allows the capturing of longitudinal characteristics before and after infection in critically ill patients. This feature can be explored by restricting patients who acquired infection during ICU stay. A typical example is the subjects with intracranial hemorrhage who developed aspiration pneumonia in ICU. Risk factors for the development of infection can be analyzed. Second, for patients who had the infection before ICU admission, the severity spectrum ranging from infection, systematic inflammatory response syndrome, sepsis, severe sepsis, and septic shock can be captured. Third, the diagnosis of sepsis with international statistical classification of diseases (ICD) code is not accurate because there are many versions of sepsis definition. To include all infection patients allows the exploration of the agreement between these definitions. Clinical studies to develop the sepsis early warning system required the whole spectrum of diseases to be included in the database (20, 21). The critical care database comprises high granularity data including laboratory findings, baseline characteristics, medications, ICD-10 code, and nursing charts, and follow-up results were integrated to make a comprehensive database. The database can be utilized for a variety of clinical study purposes, such as epidemiology of risk factors, predictive analytics, natural language processing, and subphenotype identification.

## Methods

### Study Setting and Population

The study was conducted in Zigong Fourth People's Hospital, Sichuan, China from January 2019 to December 2020, and was approved by the Ethics Committee of Zigong Fourth People's Hospital (Approval Number: 2021-014). Informed consent was waived due to the retrospective design of the study. The study complies with the Declaration of Helsinki.

All patients who transferred to any type of ICU in the hospital from January 2019 to December 2020 were potentially eligible to be included in the database. Electronic healthcare records of consecutive ICU patients with the diagnosis of infection, irrespective of the place where the infection was acquired, were included in the database. Infection was defined according to the diagnosis descriptions that contained keywords such as “infection”, “pneumonia” and “-itis”. Because the original diagnosis description was recorded in simplified Chinese, in which the above keywords were extracted *via* “Ganran” and “Yan”. Some autoimmune or connective tissue diseases such as systemic lupus erythematosis (SLE), multiple sclerosis, rheumatoid arthritis, and Sjögren's syndrome were excluded manually.

### Database Development

The critical care database was populated with data that have been acquired during routine clinical practice. Thus, the establishment of the database did not interfere with the clinical practice and was not associated with increased burden on healthcare providers, as well as risks for patients. Data were exported from several information systems including electronic healthcare records (EHR), hospital information system (HIS), laboratory information system (LIS), and critical care nursing chart system. The database was finally organized into seven tables in “.csv” format (Table 1). These data tables can be related to each other by patient ID (i.e., INP_NO or PATIENT_ID).

**Table 1:** Overview of the data tables in the critical care database.

**Table name**	**Description**
*dtBaseline.csv*	This data table contains data on baseline characteristics of individual patients. One line represents one patient entry.
*dtDrugs.csv*	This data table contains data from the HIS and it is medical order prescribed by physicians. The *datatime* represents the time of the prescription and is not necessarily the time of drug administration.
*dtICD.csv*	This data table contains ICD-10 code and diagnosis descriptions. The description was translated from Chinese words. The *Status_Discharge* column describes the status of each individual diagnosis. If a patient died on hospital discharge, *Status_Discharge* will be coded as “dead” for all diagnoses. This table can be used to compute hospital mortality.
*dtLab.csv*	Laboratory variables, as well as the reference range for each item, are listed.
*dtTansfer.csv*	The data table contains information on transferring between different departments, i.e., from the gastroenterology department to ICU.
*dtNursingChart.csv*	The nursing chart contains all kinds of recordings by bedside nurses. The progress notes were written in Chinese, which can be used for natural language processing.
*dtOutCome.csv*	The outcomes of included patients. Especially, it contains the SF-36 questionnaire, which was obtained by follow-up after being discharged home.
*datDictionary.csv*	Description for the column variables in each table.

*SF-36, Short Form Health Survey; ICU, intensive care medicine; ICD, International Statistical Classification of Diseases and Related Health Problems; HIS, Hospital Information System*.

The core table *dtBaseline* contained baseline demographics of included patients, and it can be linked to other tables by either INP_NO or PATIENT_ID. PATIENT_ID was used to identify unique patients and INP_NO was used to identify unique hospital admission.

The *dtOutCome* table was generated by manual entry during follow-up. The *Death_Date* was recorded as hours from admission. The Short-Form Health Survey (SF-36) questionnaire was applied to evaluate the functional outcome of those who survived the critical illness. The Short Form Health Survey is a 36-item, patient-reported survey of patient health, which taps eight health concepts: bodily pain, physical functioning, role limitations due to physical health problems, role limitations due to personal or emotional problems, social functioning, energy/fatigue, emotional well-being, and general health perceptions. It also includes a single item that provides an indication of perceived change in health (22). The long-term mortality followed at 1 to 2 years after discharge was added if the patients' family members were willing to provide such information. In case a patient died after hospital discharge, the date was recorded.

Different from previous similar databases such as MIMIC-III which only contain laboratory values measured during ICU stay, we included all laboratory values during the index hospitalization including those measured outside ICU (11). We believe this can help to capture the full trajectory of pathophysiological changes before and after critical illness. For example, the identification of patients with acute kidney injury (AKI) is usually challenging if baseline serum creatinine (measured before the onset of the critical illness) is not available (23). Some stamp time points of laboratory measurements are earlier than the hospital admission time because these were measured in the emergency room or outpatient visit before hospital admission.

### Deidentification

The data were deidentified before incorporating into the critical care database. The Health Insurance Portability and Accountability Act (HIPAA) protected health information identifiers including patient name, cell phone/telephone numbers, address, and any other variables that could uniquely identify the individual in structured data sources. The key variables PATIENT_ID and INP_NO were randomly assigned a unique number and the original patient ID and hospital ID were removed. Event time points were replaced with an offset value measured in hours from the hospital admission time (i.e., hospital admission time was the zero point). The original time points were removed from the dataset. Patients older than 89 years were assigned a random number from 90 to 120 for the age variable.

## Data Records

The study generated a relational database consisting of seven tables (Table 2). The database integrated comprehensive information of 2,790 patients in ICU with infection from January 2019 to December 2020. Tables are linked by identifiers such as INP_NO or PATIENT_ID. INP_NO refers to unique hospital admission and PATIENT_ID refers to a unique subject.

**Table 2:** Comparisons of clincial variables between survivors and non-survivors during hospital say.

**Variables**	**Total (*n* = 2,790)**	**Survivors (*n* = 2,629)**	**Non-survivors (*n* = 161)**	***p***
Age, median (Q1,Q3)	69.2 (56, 78.8)	69.3 (56.1, 78.8)	67.8 (54.9, 79.6)	0.768
Sex, *n* (%)				0.014
Female	1,114 (40)	1,065 (41)	49 (30)	
Male	1,676 (60)	1,564 (59)	112 (70)	
InfectionSite, *n* (%)				0.003
Abdomen	180 (6)	178 (7)	2 (1)	
Biliary	74 (3)	73 (3)	1 (1)	
Brain	22 (1)	21 (1)	1 (1)	
Intestine	40 (1)	40 (2)	0 (0)	
Liver	32 (1)	31 (1)	1 (1)	
Mediastinum	3 (0)	3 (0)	0 (0)	
Others	325 (12)	306 (12)	19 (12)	
Pancreatitis	63 (2)	60 (2)	3 (2)	
Pelvic	3 (0)	3 (0)	0 (0)	
Pneumonia	1,876 (67)	1,745 (66)	131 (81)	
Soft Tissue	71 (3)	71 (3)	0 (0)	
UTI	101 (4)	98 (4)	3 (2)	
ICU LOS (days), median (Q1,Q3)	4 (1.8, 10.1)	4 (1.8, 10.2)	2.8 (0.9, 9.9)	0.012
Hospital LOS (days), median (Q1,Q3)	11 (2.9, 22.5)	11.7 (3.2, 22.9)	3.3 (0.9, 10.4)	< 0.001

*Q1, first quartile; Q3, third quartile; ICU, intensive care unit; LOS, length of stay; UTI, urinary tract infection*.

High-granularity charted events such as progress notes, fluid intake, consciousness, vital signs, mechanical ventilator parameters, Richmond Agitation-Sedation Scale (RASS), and critical-care pain observation tool (CPOT) scores are recorded in the nursing chart table. Information from different sources might be inconsistent. For example, a drug may be prescribed by the physician as recorded in the *dtDrugs* table. However, the drug is actually not administered and thus will not be found in the *dtNursingChart* table. Our approach is to keep these tables independent for clarity because these tables reflect different sources of information and contain information for prognostic or predictive analytics. For example, the physician may prescribe analgesics for a patient on admission, but this patient actually does not experience pain or agitation and the analgesics are not actually administered. However, the presence of medical order reflects the physician's expectation and thus may contain prognostic information. The dataset is available at PhysioNet (https://physionet.org/content/icu-infection-zigong-fourth/1.0/).

### Technical Validation

Data were retrospectively extracted from the information systems in the Zigong Fourth People's Hospital. Firstly, the required data were exported from an electronic healthcare database with the assistance of an information technology technician (Zhou). The exported data were then reviewed by three expert critical care physicians (PX, LC, and ZZ). Most variables recorded in Chinese such as diagnosis description, laboratory item, and department name were translated into English. However, the progress notes from the nursing chart remained in Chinese because such information can be used for natural language processing. Some embedding features might be lost or modified when they are translated into other languges (24). In the meantime, some impossible date entries (follow-up date earlier than the discharge date), impossible values from the nursing chart (i.e., respiratory rate = 2), and outliers (i.e., tidal volume = 30) were either removed or updated after a manual check. Data were finalized and fully anonymized on August 20, 2021.

## Usage Notes

### Data Access

The critical care database is provided as a collection of comma-separated value (CSV) files. Such files can be easily processed with popular languages scripts such as PostreSQL, MySQL, R (version 4.01, The R Foundation for Statistical Computing), and MonetDB. In particular, the relational database can be easily managed with the *tidyverse* pipeline. In *tidyverse* pipeline, all packages can be fit together seamlessly and users do not need to worry about compatibility issues between different functions from different sources, and *tidyverse* scripts are easier to write, read, and understand than base R code (25). Users are required to formally request access to the database.

#### Baseline Characteristics of Included Patients

The overall mortality rate at hospital discharge was 5.8% (161/2,790). The proportion of men was higher in non-survivors than that in survivors (70 vs. 59%; *p* = 0.014). Patients with pneumonia were more likely to die than other sites of infection. However, non-survivors showed a shorter length of stay in both hospital and ICU, which was attributable to the fact that many severely ill patients chose to withdraw life-support interventions and died shortly after a few days of treatment.

**Figure 1:**
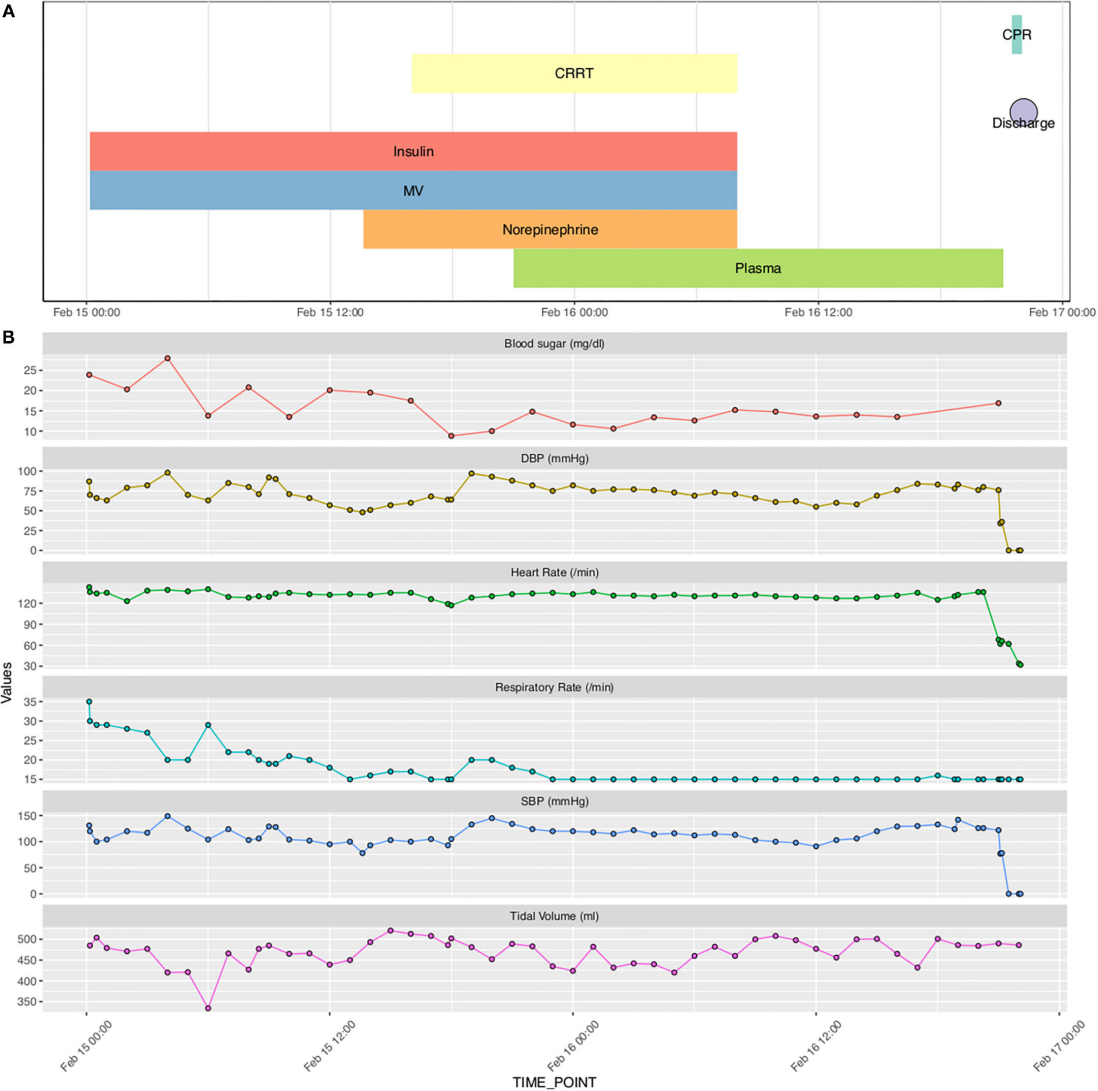
Sample data for a single patient stay in the ICU. **(A)** Chart events extracted from free texts recorded in the nursing chart; **(B)** Structured numeric data extracted from the nursing chart. CRRT, continuous renal replacement therapy; MV, mechanical ventilation; CPR, cardiopulmonary resuscitation; SBP, systolic blood pressure; DBP, diastolic blood pressure.

Sample data for a single patient stay in the ICU are shown in Figure 1. The patient was transferred to ICU and experienced septic shock. Norepinephrine was used to maintain blood pressure. Organ failures including acute kidney injury, respiratory failure, and circulatory shock occurred sequentially during the disease course. Supportive treatments such as continuous renal replacement therapy (CRRT), mechanical ventilation (MV), and vasopressor were used. However, the clinical conditions deteriorate and suffered from sudden cardiac arrest (Figure 1).

## Data Availability Statement

The datasets presented in this study can be found in online repositories. The names of the repository/repositories and accession number(s) can be found at: https://physionet.org/content/icu-infection-zigong-fourth/1.0/.

## Ethics Statement

The studies involving human participants were reviewed and approved by Ethics Committee of Zigong Fourth People's Hospital. Written informed consent for participation was not required for this study in accordance with the national legislation and the institutional requirements.

## Author Contributions

ZZ, LC, and PX conceived the idea. YZ and SY curated data. RC and WH checked the accuracy of the data. FW performed a patient follow-up. All authors contributed to the article and approved the submitted version.

## Funding

PX received funding from the RUIYI emergency medical research fund (202013), Open Foundation of Artificial Intelligence Key Laboratory of Sichuan Province (2020RYY03), and a Research project of the Health and Family Planning Commission of Sichuan Province (17PJ136). ZZ received funding from Yilu Gexin-Fluid Therapy Research Fund Project (YLGX-ZZ-2020005), Health Science and Technology Plan of Zhejiang Province (2021KY745), the Key Laboratory of Tropical Cardiovascular Diseases Research of Hainan Province (Grant No. KLTCDR-202001), and the Key Laboratory of Emergency and Trauma (Hainan Medical University), Ministry of Education (Grant No. KLET-202017). LC received funding from the Key Laboratory of Emergency and Trauma (Hainan Medical University), Ministry of Education (Grant No. KLET-202118).

## Conflict of Interest

The authors declare that the research was conducted in the absence of any commercial or financial relationships that could be construed as a potential conflict of interest.

## Publisher's Note

All claims expressed in this article are solely those of the authors and do not necessarily represent those of their affiliated organizations, or those of the publisher, the editors and the reviewers. Any product that may be evaluated in this article, or claim that may be made by its manufacturer, is not guaranteed or endorsed by the publisher.
